# Multifunctional auxetic and honeycomb composites made of 3D woven carbon fibre preforms

**DOI:** 10.1038/s41598-022-26864-x

**Published:** 2022-12-30

**Authors:** Hassan M. El-Dessouky, Chris McHugh

**Affiliations:** 1 Physics Department, Faculty of Science, Galala University, New Galala City, 43511 Egypt; 2 Composite Centre, Advanced Manufacturing Research Centre (AMRC), University of Sheffield, Sheffield, S60 5TZ UK; 3 Physics Department, Faculty of Science, Mansoura University, Mansoura, 35516 Egypt

**Keywords:** Structural materials, Composites, Mechanical properties

## Abstract

Three dimensional (3D) woven composites started to find applications in various industrial sectors, mainly in aerospace and with a potential in automotive. 3D-woven fabrics can be architected to form complex and near-net-shape preforms ready for automated composites manufacturing. The 3D-woven honeycomb fabric is designed to include additional functionality into finished composites, such as positive and negative Poisson's ratios. In this study, complex honeycomb architectures were created using various weave designs to demonstrate the effects of auxetic behaviours when manufactured into a composite structure. A Staubli 3D-weaving system equipped with Jacquard UNIVAL 100 and creel of 3072 6 k carbon fibre tows were used to weave the designed honeycomb architecture. With the aid of hard polyester foam inserts, the 3D-woven fabrics were converted to honeycomb and auxetic preforms. These preforms were infused using epoxy resin to manufacture a set of honeycomb and auxetic composite structures. In comparison with the baseline honeycomb structure, it is proven that the developed auxetic composites exhibited negative Poisson’s ratio of − 2.86 and − 0.12 in the case of tensile and compression tests respectively.

## Introduction

Multifunctional 3D woven composites have the ability to absorb energy through progressive failure, whilst maintaining gradual load profile decay beyond the failure onset^1,2^. Consequently, they are of great interest for situations where the ability to withstand crash or impact loading is a design requirement. 3D woven composites are starting to find applications in various sectors, particularly aerospace and automotive applications. Several OEMs and Tier 1 manufacturers are actively investigating these structures. In aerospace, 3D woven structures are already used in fan blades and fan casings. Development is at an early stage and there are many opportunities for improving impact performance and optimising the weight of the structure. It is important that crash structures used in vehicles like cars, buses and trains are accurately predictable and the manufacturing is repeatable. There is also an opportunity using 3D weaving to add an additional functionality into composites.

3D weaving is a specialist activity and there are very few centres capable of conducting the research needed. Textile manufacturers such as DORNIER and STAUBLI manufacture 3D weaving machines but 3D woven fabrics for composites applications are currently in their infancy. In the UK, companies such as Sigmatex UK Ltd, M Wrights & Sons and Antich & Sons have developed internal capabilities to utilise 3D weaving, but more R&D is required to deploy such technology throughout the supply chain. Recently the University of Sheffield AMRC established 3D weaving capabilities which will be used to bridge the gap and support industry.

**Figure 1:**
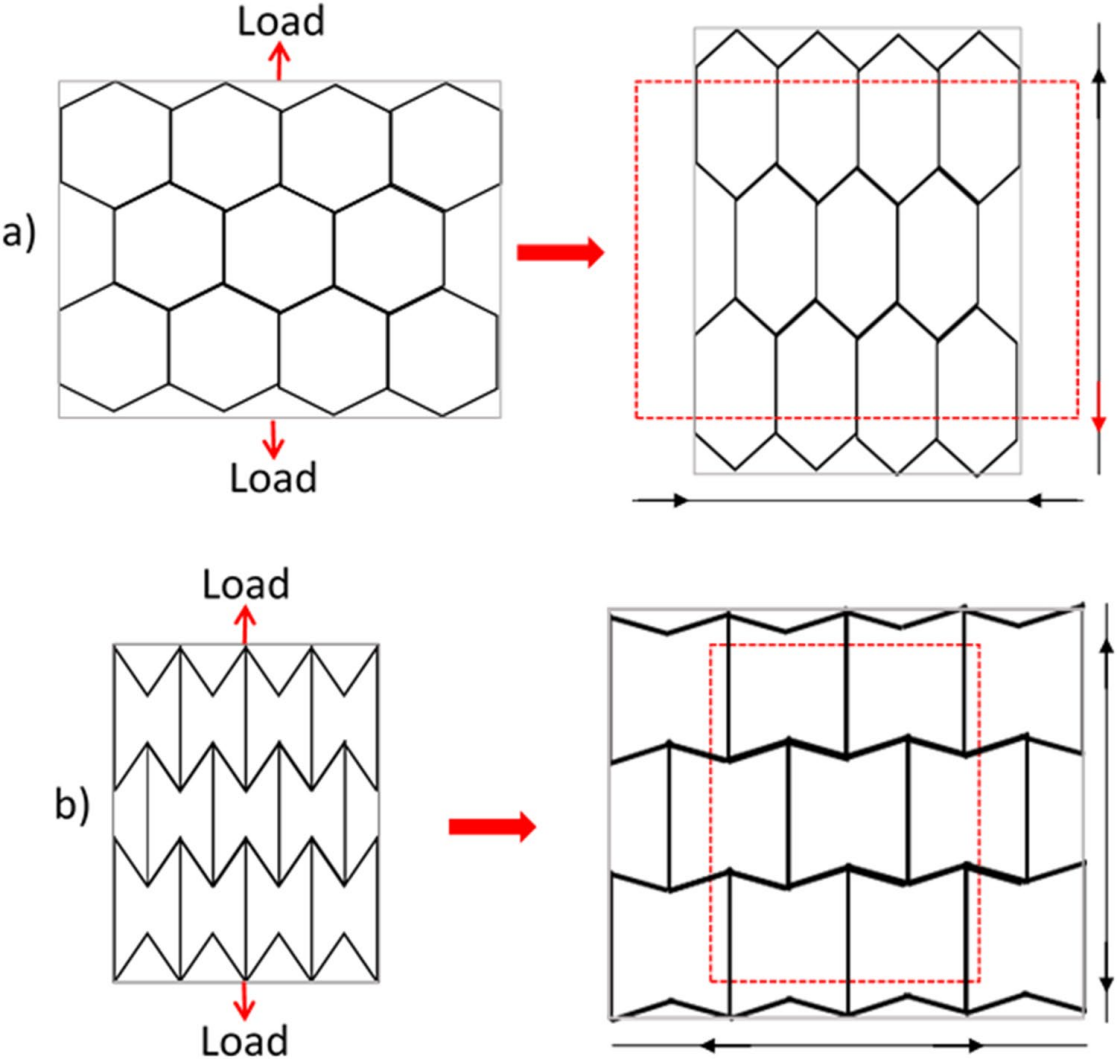
Conventional honeycomb (**a**) and auxetic (**b**) structures under tension.

3D woven preforms have the capability to demonstrate multifunctionality in the manufacture of advanced composites. One of the 3D multifunctional structures is the auxetic functionality which needs to be investigated and demonstrated to industry. This could be in the form of expandable honeycomb type structures^3^, which could be woven and tested to show capability and potentially improved mechanical performances with high damage tolerance such as crash, compression and impact. Figure 1 explains what the auxetic structure is compared to the conventional honeycomb structure in terms of its geometry, i.e., an auxetic material exposed to tension would increase in dimensions in the direction that is lateral to an applied tensile force. An auxetic structure has several advantages in a crash situation for example good energy absorption, however, the repeatable manufacture of an auxetic structure with a predictable behavior needs further work^4^.

Poisson’s ratio, which is the ratio of the strain normal to the applied load to the extension strain (or axial strain) in the direction of the applied load. Poisson's ratio ($\nu$) of standard material can be expressed as:

$${\upnu } = - \frac{{{\upvarepsilon }_{{\text{t}}} }}{{{\upvarepsilon }_{{\text{l}}} }}\;where\;{\upvarepsilon }_{{\text{t}}} = \frac{{\Delta {\text{T}}}}{{{\text{T}}_{{\text{o}}} }}\;and\;{\upvarepsilon }_{{\text{l}}} = \frac{{\Delta {\text{L}}}}{{{\text{L}}_{{\text{o}}} }}$$

where, ε_t_ = transverse strain, ε_l_ = longitudinal or axial strain, ∆L = change in length, *L*_o_ = initial length, ∆T = change in width and *T*_o_ = initial width.

Most conventional materials show positive Poisson's ratio (PPR) under tensile loads because they exhibit positive longitudinal and negative transverse strains, but smart materials like auxetics behave oppositely and show negative Poisson's ratio (NPR).

It is known that conventional materials such as rubber and metals laterally contract when stretched and laterally expand when compressed in the longitudinal direction; such materials have a PPR. In contrast, there are some special materials which possess a NPR which laterally expand when stretched or laterally shrink when compressed in the longitudinal direction. The materials with NPR are also called ‘auxetics’, which originated from the Greek word ‘auxetos’ meaning ‘that which may be increased’^5^. Auxetics could be materials and/or structures, they have been investigated in the literature from different perspectives such as developing materials and structures, comparing behaviours and testing performances.

In comparison with conventional materials, auxetic structures have many improved properties. They have higher shear modulus, hence better shear resistance. Auxetic materials have enhanced indentation/impact resistance and energy absorbance properties. When conventional material is subjected to an impact force, the material moves away from the impact point, but exhibiting the opposite behaviour, the auxetic material flows towards to impact point, which makes the auxetic materials harder to be indented. They also have other advantages, such as enhanced fracture toughness, improved crack growth resistance and higher damping resistance. Due to these advantages, auxetic composite structures could find suitable applications in high value manufacturing, such as aerospace and automotive sectors. The disadvantage of auxetic composites is that they may be difficult to manufacture on a large scale ^5^, but such difficulty has been challenged in this work.

Many studies have been conducted to develop and investigate new auxetic structures and materials based on different material scales. The examples include auxetic fibers^6,7^, auxetic fabrics^8,9^, auxetic foams^10,11^, and auxetic composites^12,13^. Auxetic woven-composite structures are investigated in this project. Zhou et al.^14^ developed auxetic composites made of 3D orthogonal woven textile and polyurethane foam. They prove that the auxetic composites exhibited NPR and behave more like damping material with lower compression stress, while the non-auxetic composites behaves more like stiffer material with higher compression stress. In another study^15^ 3D-woven structures were produced and the effect of float length of ground weave and binding yarn on auxeticity of the fabric was investigated. A set of different 3D orthogonal woven structures were produced on a rapier dobby loom by changing the float length in the ground weave and binding yarns. The results showed that the 3D-woven materials with equal and maximum float length of ground weave and binding yarn showed greater auxetic behavior. Also, the impact energy absorption of the developed composites was found to increase with the increase in float length, justifying that the structures are auxetic and possess NPR. Zulifqar and Hu^16^ reported that the woven fabric could be auxetic through a combination of loose weave and tight weave in the same structure. They showed that the developed fabrics exhibit NPR effect in both weft and warp directions in a large range of tensile strain.

In this work, a Staubli 3D Weaving System was utilised, including Unival jacquard to weave 3D honeycomb fabrics using Toray T300-6 k carbon fibres fed from warp and weft directions. With the aid of polyester foam, the developed 3D-woven fabrics were converted to two different preforms: conventional honeycomb and new auxetic structures. The preforms were infused using epoxy resin to manufacture large composite structures investigated in this study. Tensile and compression tests were carried out to assess the functionality of honeycomb and auxetic composite structures through their Poisson’s ratio measurements.

## Materials

Carbon fibre (CF), thermoset resin system and hard PET foam were used to preform and manufacture composite structures. Their grade and properties are given Table 1:

**Table 1:** Materials’ properties.

Material	Grade	Density (g/cm^3^)	Modulus (GPa)	Tensile strength (MPa)	Poisson’s ratio
Fibre	T300-6 k-CF	1.76	230 (tensile)	3530	0.26
Resin	T-Prime 130–1	1.11	2.81 (tensile)	–	0.30
Foam	Divinycell P150	0.15	0.040 (shear)	2.45	–

According to the resin manufacturer, the *T*-Prime 130–1 was mixed at 100/27 by wt% of resin/hardener to infuse the dry preforms.

### Weave design and fabric manufacturing

**Figure 2:**
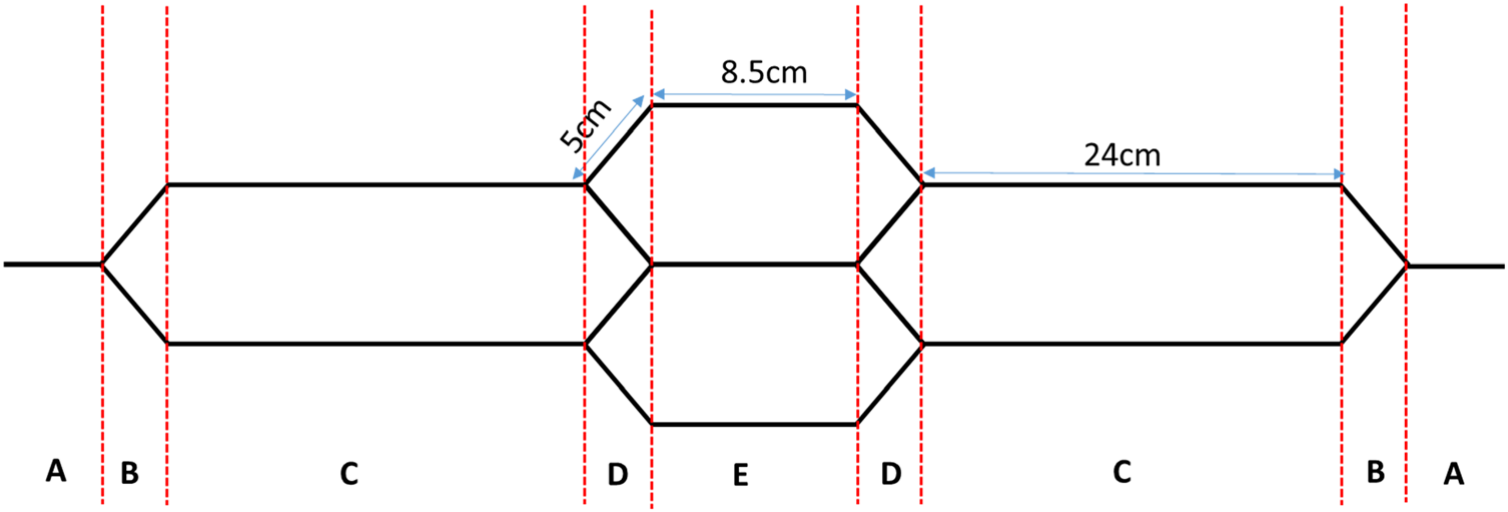
Honeycomb structure, (**A**,**B**,**C**,**D**,**E**) are plain weaves of different number of layers; 1-layer (**A**), 2-layer (**B**, **C**), 4-layer (**D**) and 3-layer (**E**).

EAT weave-design software was used to design a complex honeycomb structure. A schematic diagram of the honeycomb design suggested in this study is shown in Fig. 2. The unit cell of this honeycomb structure (Fig. 2) consists of a number of plain weaves of different number of layers; single-layer weave (A), two-layer weave (B, C), four-layer weave (D), and three-layer weave (E).

**Figure 3:**
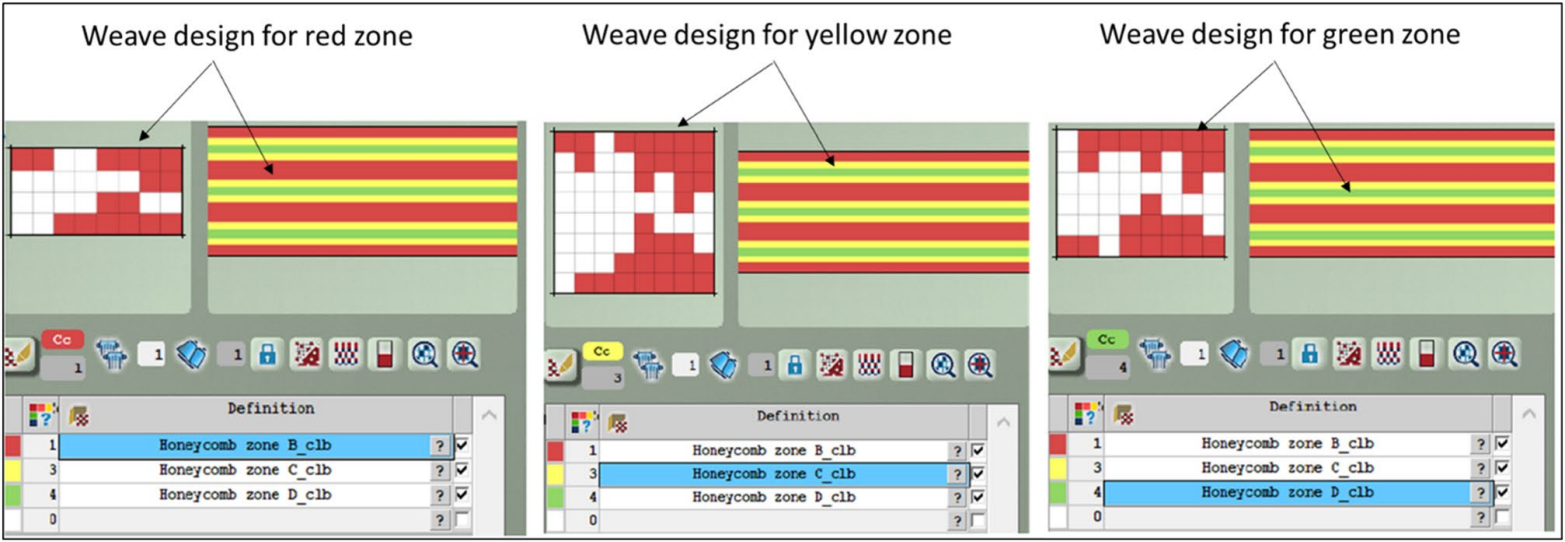
Weave designs used for the repeat zones (Red, Yellow and Green) given in Table 2 using JC5 output file from EAT software.

Using the EAT software, a colour coding system has been firstly allocated and then assigned to the different weave designs selected to form the suggested honeycomb structure. Table 2 below presents the number of picks and pick density of the different zones defined within the structure designed. Figure 3 shows the EAT assigned weaves (red, yellow and green zones) include JC5 file of the honeycomb design installed for the Jacquard UNIVAL 100.

**Table 2:** Weave design parameters of honeycomb structure.

Zones	Pick range	No. of picks	Density (pick/cm)	Colour
1	1–82	81	6	Red
2	83–138	55	10	Yellow
3	139–210	71	8	Green
4	211–266	55	10	Yellow
5	267–428	161	6	Red
6	429–484	55	10	Yellow
7	485–556	71	8	Green
8	557–612	55	10	Yellow
9	613–770	157	6	Red
10	771–826	55	10	Yellow
11	827–898	71	8	Green
12	899–954	55	10	Yellow
13	955–1032	77	6	Red

**Figure 4:**
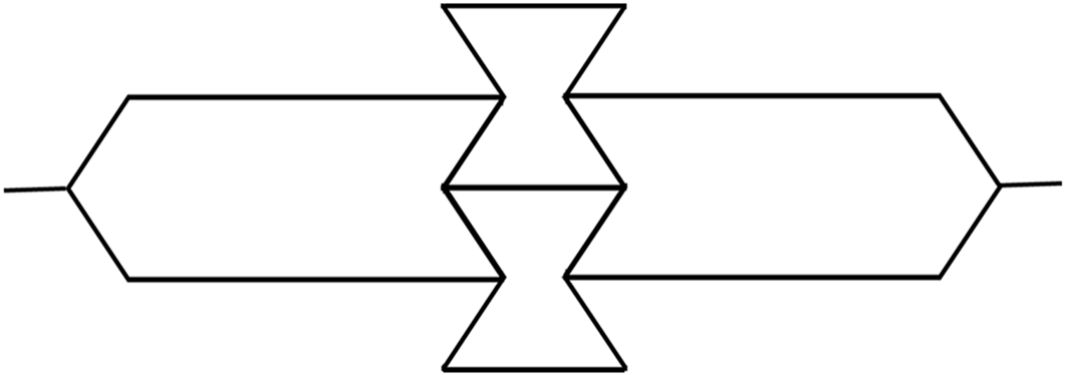
Auxetic structure.

The main goal of this research is to demonstrate a 3D woven composite structure that can exhibit a smart functionality such as an auxetic structure of NPR. The honeycomb structure designed in this study (Fig. 2) is converted to the auxetic one as shown in Fig. 4.

**Figure 5:**
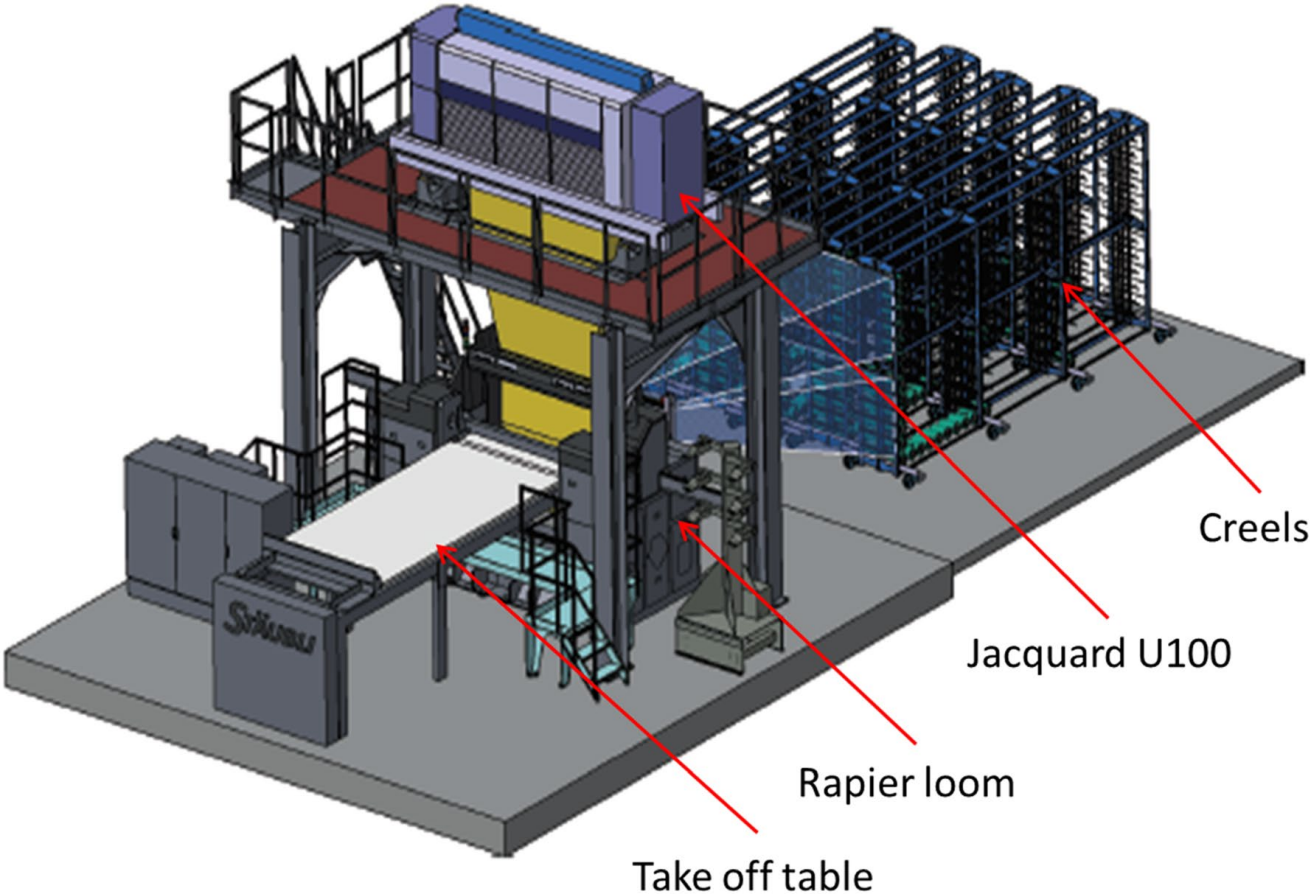
A schematic of the 3D weaving system.

**Figure 6:**
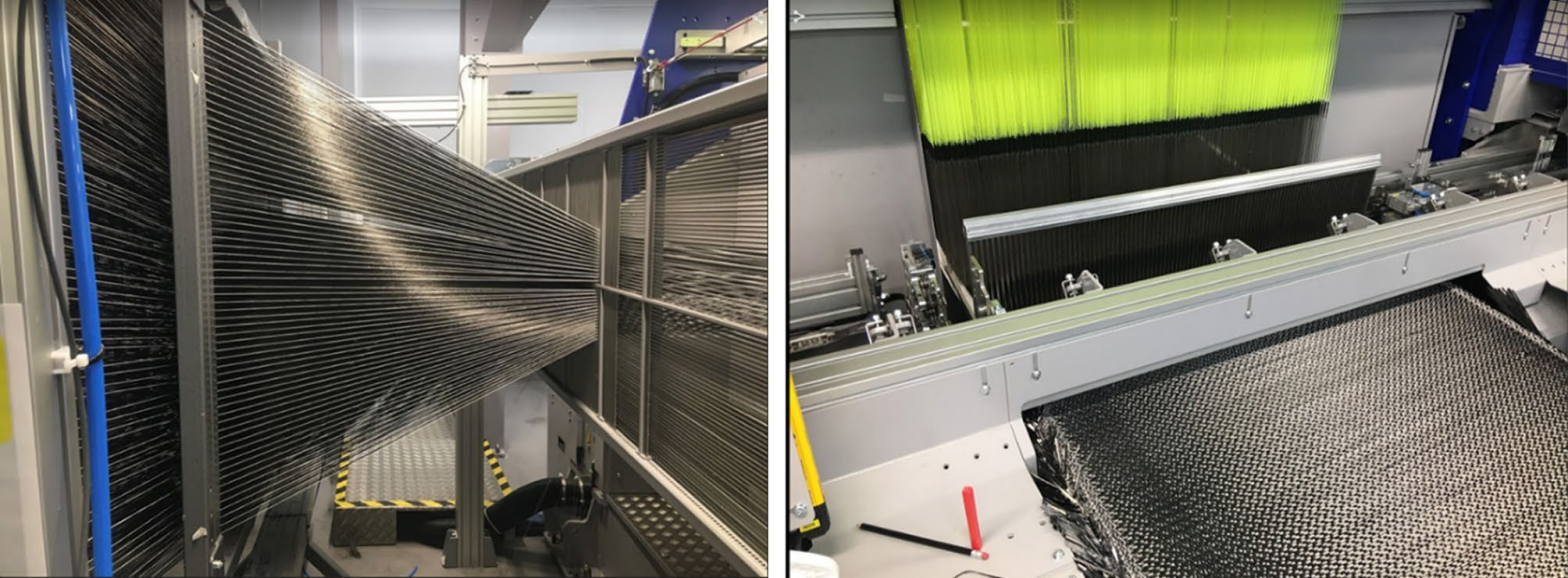
The 3D weaving machine in operation with fibres loaded at the back (left) and fabric produced at the front (right) of the loom.

The 3D weaving system (creel, Jacquard, loom and horizontal take off table) was used to produce the honeycomb fabric. The 3D weaving system (Fig. 5) was threaded with 3072 carbon fibre tows through the warp direction and the same fibre was also used through the weft direction. 16 ends per dent was drawn in through the reed. 128 ends (64 from each side) of the 3072 ones were loaded with polyester (PET) yarn and used as selvage catch cord to lock both edges of the woven fabric (Fig. 6).

**Figure 7:**
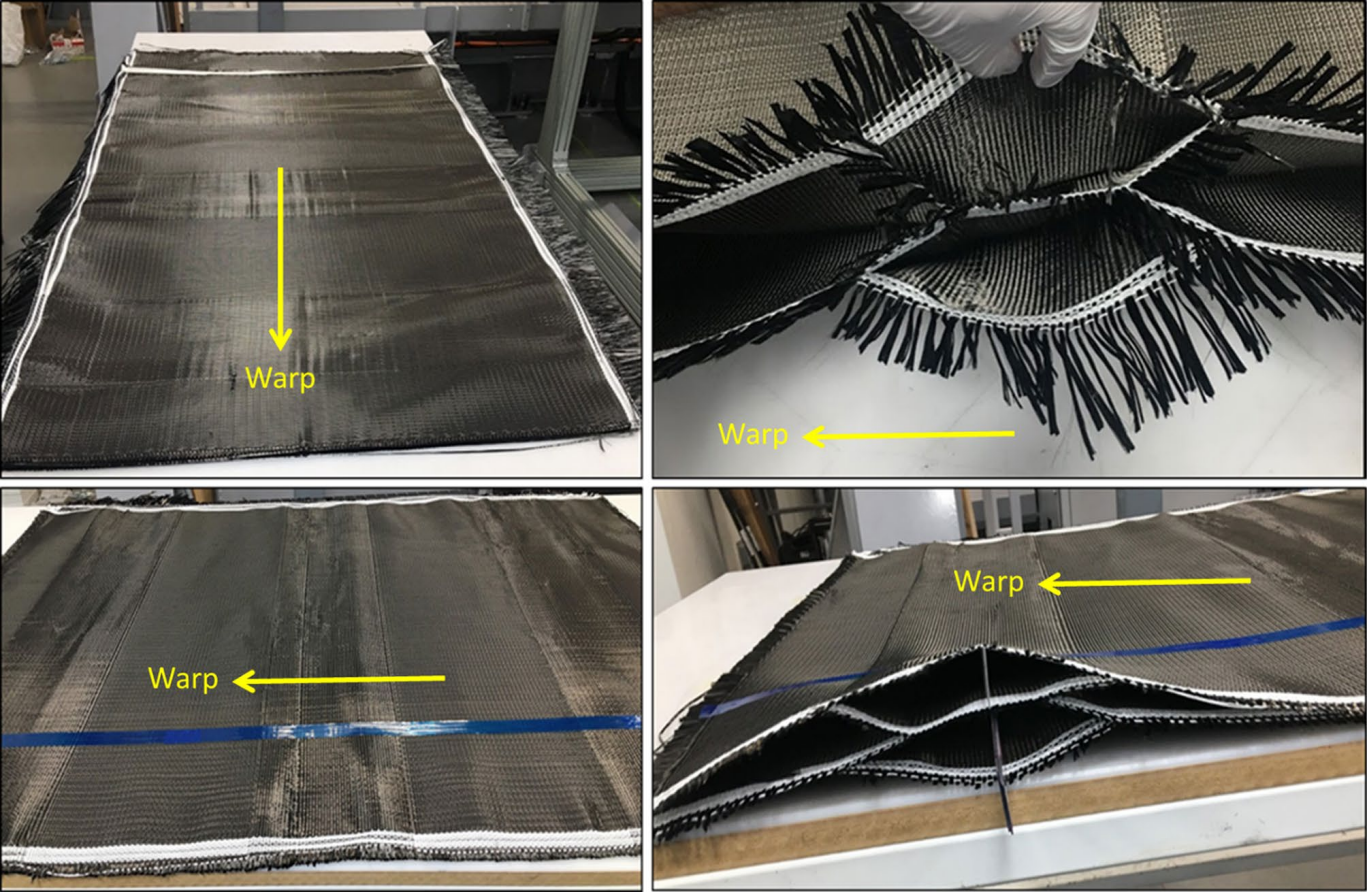
Samples of 3D woven honeycomb fabric.

According to Table 2, samples of 3D-woven fabrics of three different pick densities (6, 8 and 10 pick/cm) were manufactured. Figure 7 demonstrates a selection of photos of the honeycomb fabric produced, blank samples on the left and open cross-sections on the right.

### Dry fibre preforming and testing auxetic functionality

**Figure 8:**
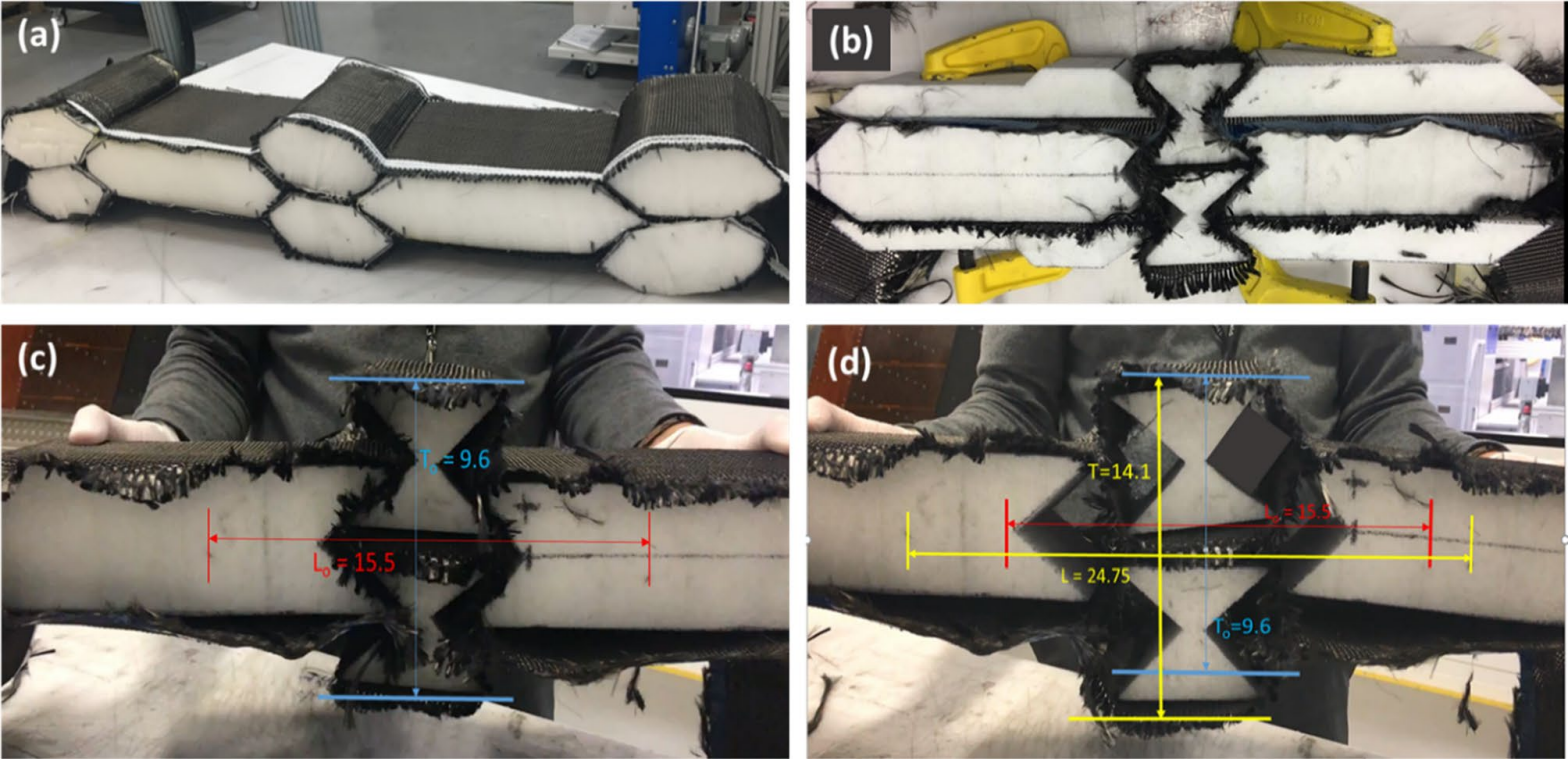
Dry fibre honeycomb (**a**) and auxetic (**b**) preforms. Length and height measurements at the start (**c**) and the end (**d**) of the manual testing of auxetic preform.

In order to minimise errors, trials and to save materials, a soft foam insert/core was used to preform the woven fabric into the honeycomb and auxetic structures prior to resin infusion. The dry fibre preform structure was made to test its formability and functionality in particular the auxetic one. The foam was cut to the relevant shapes and was inserted into the fabric pockets. The honeycomb structure (Fig. 8a) achieved its targeted preform shape relatively easily, but the auxetic preform (Fig. 8b) needed some additional supports (in the form of *G*-clamps) in order to hold the shape.

As a dry preform, the functionality of the auxetic structure was tested to confirm its negative Poisson’s ratio. Snapshots (Fig. 8c and Fig. 8d) were captured during the manual tensile testing. The longitudinal and transverse parameters such as the initial length (*L*_o_) and height (*T*_o_) were measured and highlighted on Fig. 8c,d. The units of measurements are ignored here as the strain is dimensionless and such measurements were taken online using a virtual ruler. The longitudinal and transverse strains and then Poisson’s ratio were calculated and listed in Table 3. The Poisson’s ratio is found to be negative (− 0.78) which confirms that the preform is showing auxetic behaviour.

**Table 3:** Measured displacements, strains and Poisson’s ratio of dry auxetic preform.

∆L	∆T	${{\varvec{\varepsilon}}}_{{\varvec{l}}}$	${{\varvec{\varepsilon}}}_{{\varvec{t}}}$	${\varvec{\nu}}$
9.25	4.5	0.60	0.47	− 0.78

### Composite’s manufacturing

**Figure 9:**
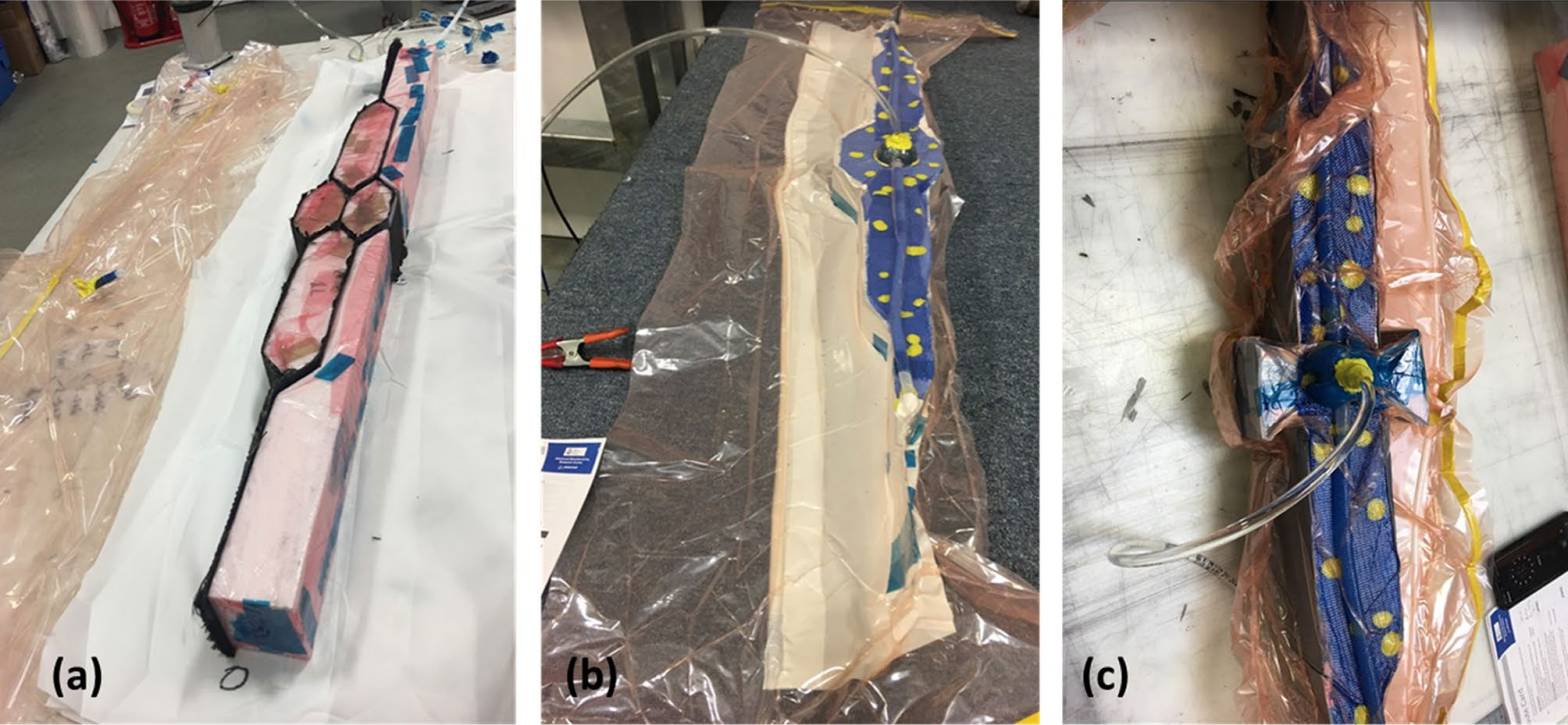
Bagging process of honeycomb preform (**a**) and resin infusion of honeycomb (**b**) and auxetic (**c**) assembles.

Due to the high complexity of the woven structures, the resin infusion and vacuum bagging method was employed in this study. To avoid squashing and compressing the soft foam, used above in Fig. 8 during the vacuuming process, alternative hard and high-density PET foam (Divinycell P150) was used to preform the honeycomb structure prior to infusion. The foam inserts were wrapped with release film to ease demoulding after curing. Figure 9a,b,c shows an example of bagging process of honeycomb preforms including the resin infusion of both honeycomb and auxetic structures. An infusion mesh or a resin flow assist material (blue) was used to encourage flow in particular across the preform as shown in Fig. 9.

**Figure 10:**
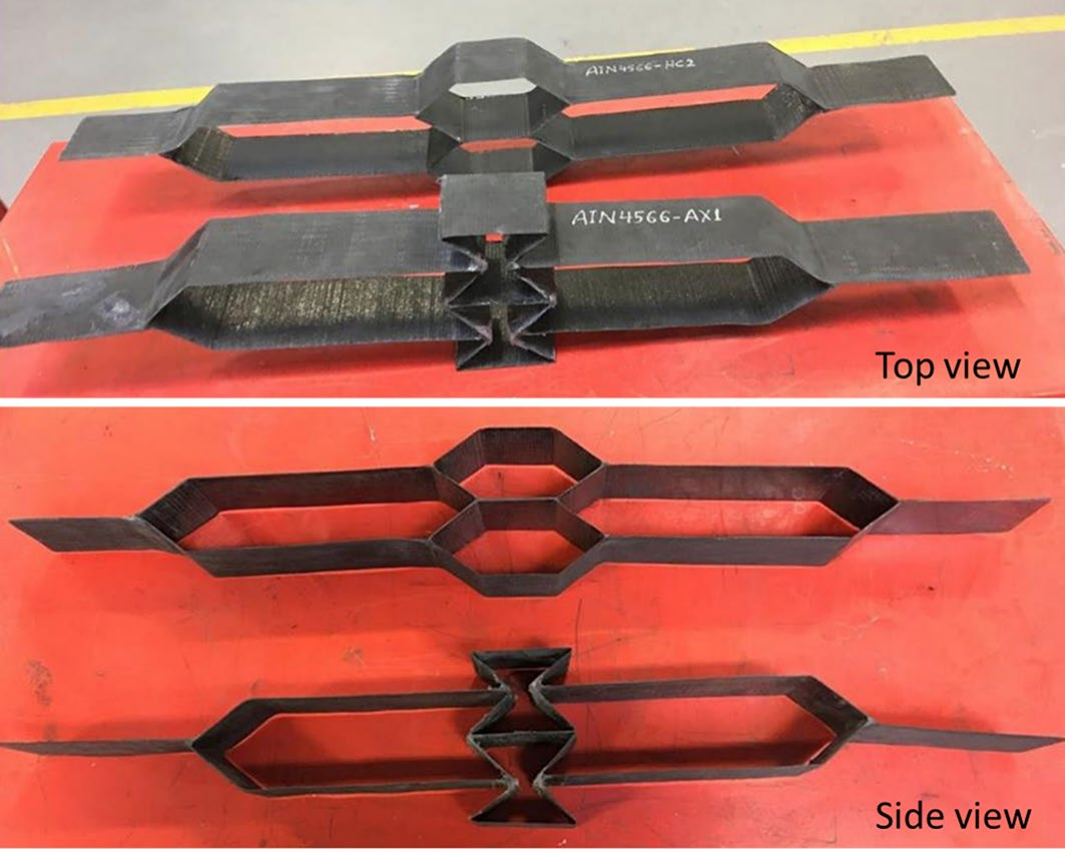
Cured honeycomb (top) and auxetic (bottom) composite structures.

Gurit *T*-Prime 130–1 resin and hardener were used to infuse the woven preforms produced in this research. The mixing ratio of resin to hardener used in these infusions was 100:27 by weight, as prescribed by the manufacturer’s TDS. Table 4 gives the mixing ratio used in grams. After degassing the mixture for 10 min, the infusion took place and was completed in around 30 min. Subsequently, the assembly was moved to a preheated oven and cured at 60 °C for 3 h. Figure 10 demonstrates a selection of the manufactured honeycomb and auxetic composite structures.

**Table 4:** Resin and hardener mixing ratios.

Material	Mixing ratio (Parts by weight)	Quantity (g)
*T*-Prime 130–1 Resin	100	800
*T*-Prime 130–1 Hardener	27	216

## Results and discussion

Mechanical tests were carried out to determine the Poisson’s ratio for the honeycomb and auxetic composite structures manufactured in this study. Despite the conventional samples such as flat and cylindrical coupons, there are no standard methods available to determine Poisson’s ratio of such complicated structures developed in this research. Instron testing machines were used to have a good control and determine the force–displacement graphs precisely. The two composite structures are subjected to tensile and compression tests, the results of which are detailed in the following sections.

### Tensile test

**Figure 11:**
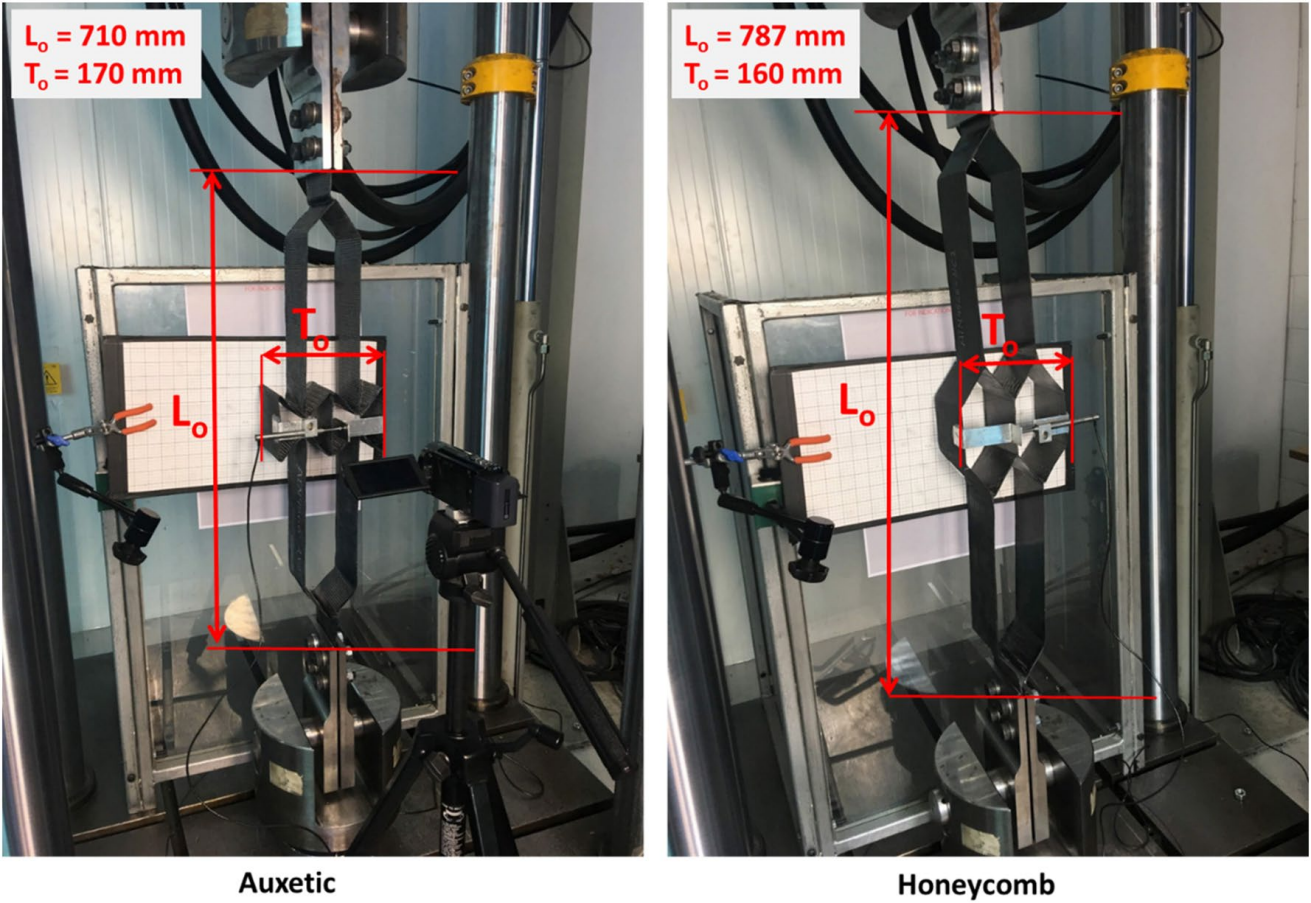
Tensile test setup of auxetic (left) and honeycomb (right) structures.

**Figure 12:**
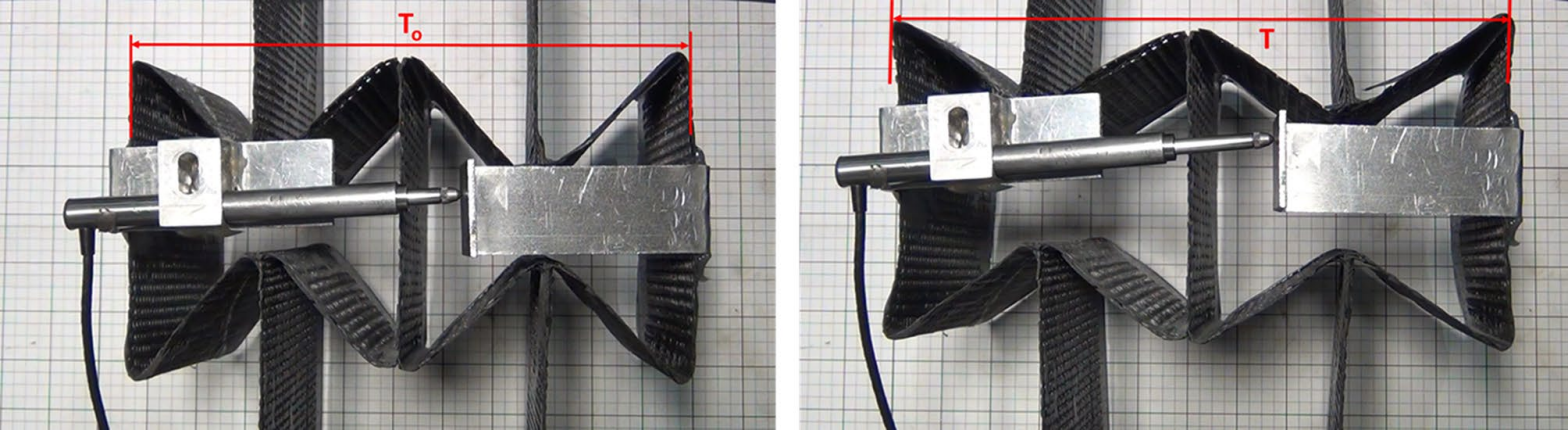
The start (left) and end (right) positions of tensile test for the auxetic structure.

Prior to testing, the length and height of the auxetic and honeycomb samples were measured as shown and given in Fig. 11. A transducer was used to ensure precise and online measurement of the transverse displacement during the test. The test repeats were recorded and two screenshots were captured to determine the initial and final transverse displacements. In the case of the auxetic structure, Fig. 12 shows the start (left) and end (right) positions of the tensile test.

**Figure 13:**
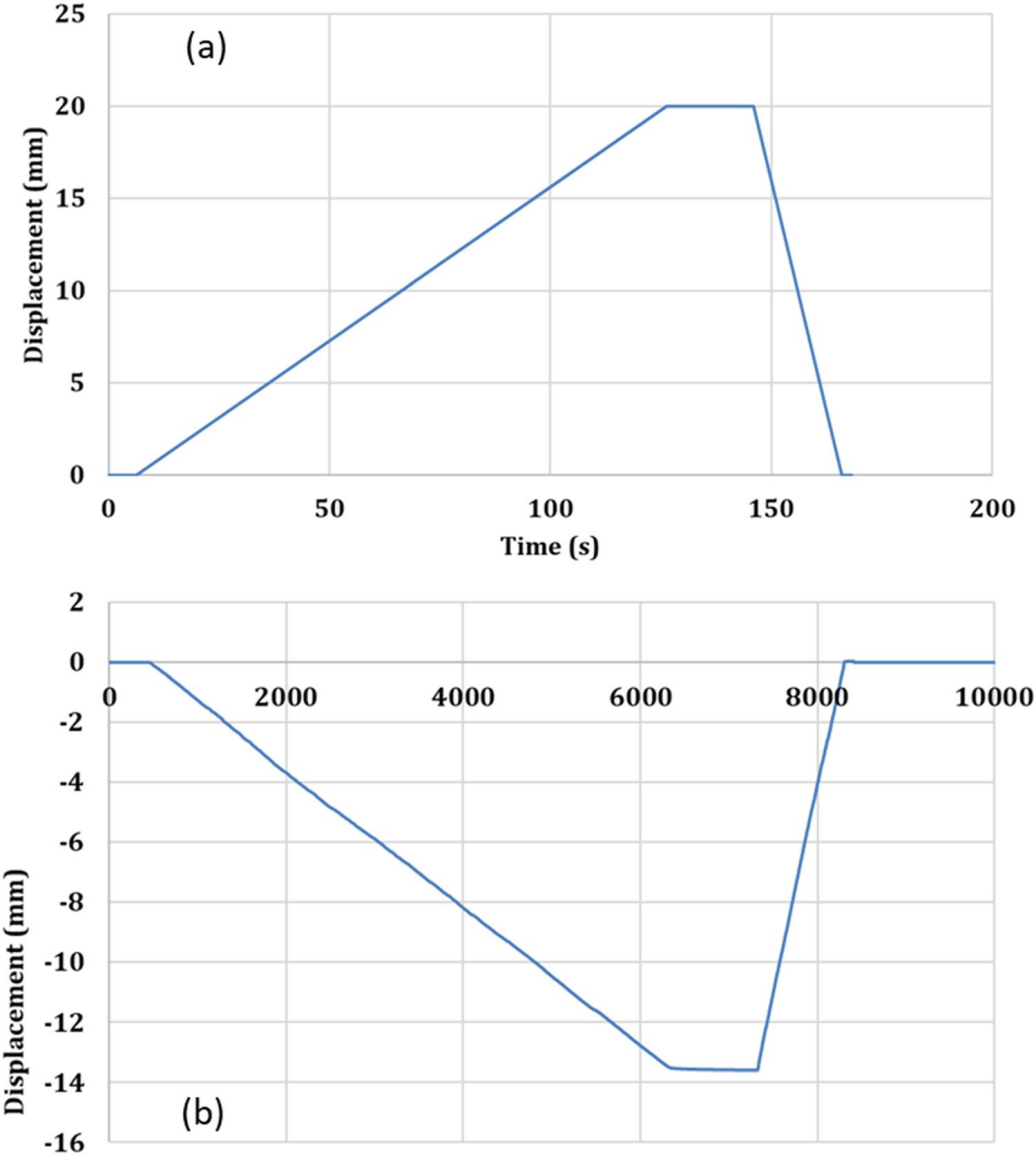
Longitudinal (**a**) and transverse (**b**) displacements recorded in the case of tensile test of auxetic structure.

Figure 13a,b shows the maximum longitudinal (L) and transverse (T) displacements of the auxetic structure recorded during the test. Due to the complexity and rigidity of the tested structure, it is noticed that the transducer has been slightly deviated from the original position at the start of the test (Fig. 13). To overcome this misalignment, the measurements of the transverse displacement were also recoded from the grid background (graph paper). From the measurements made and the figures above, Table 5 listed the measurements obtained. It is found that the Poisson’s ratio of the tested structure is − 2.86, i.e. the auxetic composite exhibited NPR in the case of the tensile testing.

**Table 5:** Measured displacements, strains and Poisson’s ratio of auxetic composite in the case of tensile test.

∆L	∆T	${{\varvec{\varepsilon}}}_{{\varvec{l}}}$	${{\varvec{\varepsilon}}}_{{\varvec{t}}}$	${\varvec{\nu}}$
20	13.52	0.028	0.080	− 2.86

**Figure 14:**
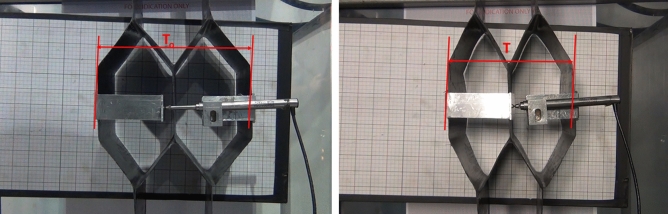
The start (left) and end (right) positions of tensile test for the honeycomb structure.

**Figure 15:**
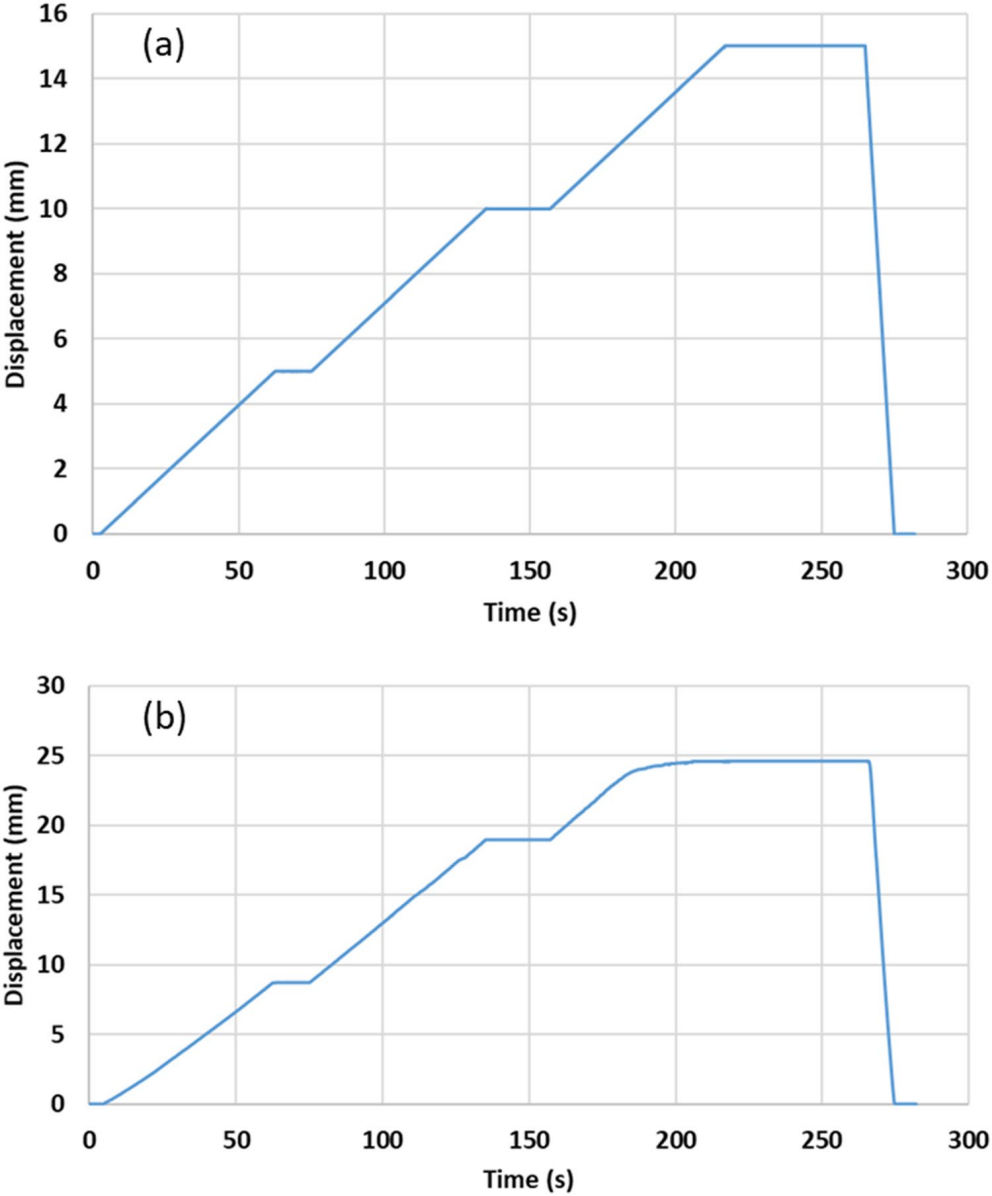
Longitudinal (**a**) and transverse (**b**) displacements of the honeycomb structure recorded during the test.

In the case of the honeycomb structure, Fig. 14 shows the start and end positions of the tensile test. The longitudinal and transverse displacements of honeycomb structure recorded during the tensile test are shown in Fig. 15a,b and the Poisson’s ratio results are given in Table 6. It is found that the Poisson’s ratio of the tested structure is 8.10, i.e. as expected the honeycomb composite exhibited PPR in the case of the tensile testing.

**Table 6:** Measured displacements, strains and Poisson’s ratio of honeycomb composite in the case of tensile test.

∆L	∆T	${\varepsilon }_{l}$	${\varepsilon }_{t}$	$\nu$
15	− 24.61	0.019	− 0.154	8.10

### Compression test

**Figure 16:**
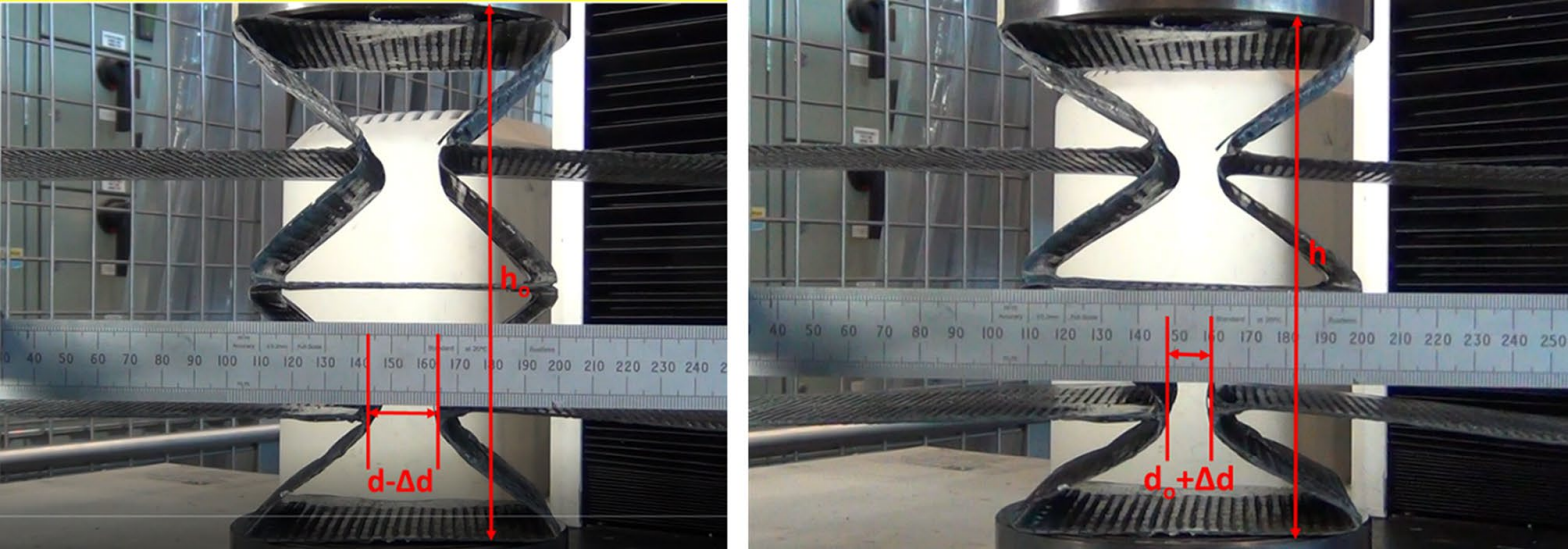
The start (left) and end (right) positions of compression test for the auxetic structure.

In the case of compression test setup, a ruler was used as an indicator of the transverse displacement instead of the transducer used in the tensile test. Different symbols were used in this test due to the change of the load direction. For the auxetic sample, the original length is noted d_o_ which measured 710 mm whilst the original height h_o_ measured 170 mm. The dimensions of d_o_ and h_o_ for the honeycomb were 787 mm and 160 mm respectively. As shown in Fig. 16, a small section was highlighted on the ruler to measure the displacement in the longitudinal direction. Figure 16 shows the start and end positions of the compression test for the auxetic structure. Table 7 gives the measured displacements, strains and Poisson’s ratio of auxetic composite. It is found that the Poisson’s ratio of the tested auxetic structure is − 0.12, i.e. the auxetic composite also exhibited NPR in the case of the compression testing. It is proven that the auxetic composite structure revealed NPR under tensile and compression loads.

**Table 7:** Measured displacements, strains and Poisson’s ratio of auxetic composite in the case of compression test.

∆h	∆d	${{\varvec{\varepsilon}}}_{{\varvec{l}}}$	${{\varvec{\varepsilon}}}_{{\varvec{t}}}$	${\varvec{\nu}}$
− 20.04	− 10.1	0.014	0.118	− 0.12

**Figure 17:**
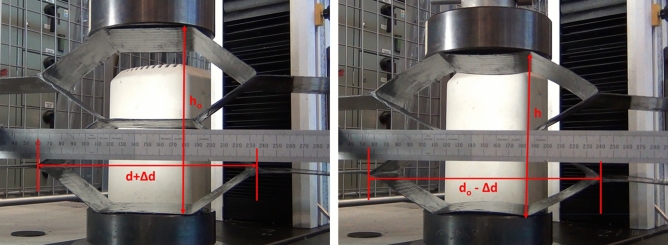
The start (left) and end (right) positions of compression test for the honeycomb structure.

In terms of the honeycomb structure, Fig. 17 shows the start and end positions of compression test. Table 8 gives the measured displacements, strains and Poisson’s ratio of honeycomb composite. It is found that the Poisson’s ratio of the tested honeycomb structure is 0.11, i.e. the honeycomb composite also exhibited PPR in the case of the compression testing.

**Table 8:** Measured displacements, strains and Poisson’s ratio of honeycomb composite in the case of compression test.

∆h	∆d	${{\varvec{\varepsilon}}}_{{\varvec{l}}}$	${{\varvec{\varepsilon}}}_{{\varvec{t}}}$	${\varvec{\nu}}$
− 20.06	10	− 0.125	0.118	0.11

In summary, it is found that the auxetic composite structure exhibited NPP (− 2.86 & − 0.12), whereas the honeycomb structure showed PPR (8.10 & 0.11) under both testing mechanisms (tensile and compression). But in the case of the tensile test, the Poisson’s ratio obtained for both structures are found to be out of the normal range of standard materials (− 1 to 1) which may be due to the specific structures developed in this work.

## Conclusion

3D honeycomb structures were successfully woven and the use of a supportive core material (in this case foam) was needed to allow the dry 3D woven fabrics to be preformed and resin infused. As a dry fibre, the auxetic preform was manually tested and its auxetic functionality was successfully proven. Dry preforms (honeycomb and auxetic) were infused using epoxy resin then the cured honeycomb and auxetic composites were successfully tested using tensile and compression tests. The honeycomb structure exhibited positive Poisson’s ratios (PPR) in both testing directions (tensile and compression), but the auxetic structure demonstrated a negative Poisson’s ratio (NPR) and thereby exhibited smart functionality. The concept of 3D woven smart functionality composites is proven, and multifunctional 3D woven composites are demonstrated. The values of Poisson’s ratio obtained for both structures are found to be outside the range of conventional materials in the case of the tensile test.

Future work is recommended to manufacture generic panels or demonstrators made of honeycomb/auxetic composites and investigate their mechanical performance through different responses such as impact and crash tests. Furthermore, the very high Poisson’s ratio for the honeycomb structure will be explored further to see if this can be exploited in new applications.

## Supplementary Information

Supplementary Information.

## Data Availability

The raw data of tests carried out in this study is available upon request and here it is a link for it. https://galalauni-my.sharepoint.com/:u:/g/personal/h_el-dessouky_gu_edu_eg/EYDA59Z5qIFFi5TMy7ojhwwBk3HBbKsli6CgsxejJpz_nQ?e=IkEtdb.
